# A Network Coverage Information-Based Sensor Registry System for IoT Environments

**DOI:** 10.3390/s16081154

**Published:** 2016-07-25

**Authors:** Hyunjun Jung, Dongwon Jeong, Sukhoon Lee, Byung-Won On, Doo-Kwon Baik

**Affiliations:** 1 Department of Computer and Radio Communications Engineering, Korea University, Anam-dong, Seongbuk-Gu, Seoul 136-701, Korea; darkspen@korea.ac.kr; 2 Department of Statistics and Computer Science, Kunsan National University, Miryong-dong, Gunsan-si, Jeollabuk-do 573-701, Korea; bwon@kunsan.ac.kr; 3 Department of Biomedical Informatics, Ajou University School of Medicine, Woncheon-dong, Yeongtong-gu, Suwon 433-721, Korea; leha82@ajou.ac.kr; 4 Department of Computer Science and Engineering, Korea University, Anam-dong, Seongbuk-Gu, Seoul 136-701, Korea

**Keywords:** network coverage, Sensor Registry System, sensor information

## Abstract

The Internet of Things (IoT) is expected to provide better services through the interaction of physical objects via the Internet. However, its limitations cause an interoperability problem when the sensed data are exchanged between the sensor nodes in wireless sensor networks (WSNs), which constitute the core infrastructure of the IoT. To address this problem, a Sensor Registry System (SRS) is used. By using a SRS, the information of the heterogeneous sensed data remains pure. If users move along a road, their mobile devices predict their next positions and obtain the sensed data for that position from the SRS. If the WSNs in the location in which the users move are unstable, the sensed data will be lost. Consider a situation where the user passes through dangerous areas. If the user’s mobile device cannot receive information, they cannot be warned about the dangerous situation. To avoid this, two novel SRSs that use network coverage information have been proposed: one uses OpenSignal and the other uses the probabilistic distribution of the users accessing SRS. The empirical study showed that the proposed method can seamlessly provide services related to sensing data under any abnormal circumstance.

## 1. Introduction

The Internet of Things (IoT) is a large-scale network of physical objects, including microcomputers and sensing nodes, that provides users with much better personalization services. Objects such as RFIDs, sensors, actuators, mobile phones, smart cars, and electronic devices are all interconnected via the Internet [1]. The IoT allows these heterogeneous objects to gather, combine, and exchange data. As a good example of these IoT-based services, SFpark (sfpark.org) has provided useful parking information since about 8200 car parking space sensor devices were installed in San Francisco. Every automobile driver can reduce the time wasted in finding an available parking space by using the SFpark application. In this case, the IoT-based service makes life very convenient.

To embody its services, the wireless sensor network (WSN) is used as the core infrastructure of the IoT. In the WSN, various types of sensed data are collected from multiple, heterogeneous sensors, e.g., temperature, illumination, humidity, sound, pressure, and vibration sensors, in real time. Then, these sensing data are combined and used to provide better services. For example, as mentioned above, the sensors located in about 8200 parking lots in San Francisco frequently collect information regarding the available parking spaces, and then send the information to an agent operating in a computer server that collates all the information and detects an available parking space near a user who requests this information. Finally, the available parking space is shown to the user on the SFpark application display. However, the IoT is likely to have different WSNs [2,3]. Each WSN stores and distributes data in a different format. For instance, the standard temperature unit in WSNs located in the US is Fahrenheit, while that in Canada and Mexico is Celsius. Thus, when sensed data are exchanged between different WSNs, interoperability problems occur. To overcome this barrier, depending on the situation, Fahrenheit must be converted to Celsius, or vice versa.

Currently, a Sensor Registry System (SRS) is used to resolve the interoperability problem. Many types of sensor information named sensor metadata are registered at the SRS. The sensor metadata are used to interpret, recognize, and process the meanings of sensed data [4].

Recently, to achieve a “safe” city, an IoT-based service was constructed in Gimpo-si near Seoul in Korea. Many sensors were attached to street lamps, security lights, closed-circuit television cameras (CCTVs), underground facility management systems controlling water/sewage pipes, electricity cables, communication lines, gas pipes, control centers of parks and apartment complexes, and even next-generation traffic information systems. In this service, the sensed data are exchanged among the heterogeneous sensors, and incidents or crimes can be efficiently predicted and thus prevented. This IoT-based system makes use of SRS to address the interoperability problem of sensed data. To enable issuing of advanced warnings about dangerous sinkholes on roads, sensors are located in subways and water/sewage pipes. To enable the prediction of a gas explosion, sensors are installed in the sectors and districts of the city. All the sensed data transmitted by the sensors located around subways and water/sewage pipes and by gas detection sensors is registered in the SRS. The mobile devices of users walking along a road regularly access and obtain the relevant sensed information stored in the SRS and attempt to interpret the meaning of the sensor data they receive.

If a user passes through dangerous areas, for example, where they could encounter sinkholes, or through a dangerous gas leak area, the user’s mobile device should predict their next position on the road relative to the current position. First, the mobile device can obtain the GPS information, using which the user’s past position is tracked. Second, based on the previous path information, several candidates for the next position can be predicted. Finally, from the current position, if one of the candidate positions was frequently selected by many other persons in a particular time interval, it is determined as the user’s next position. In this way, Path Prediction-based SRS [5] (PP-SRS) is used for receiving the relevant sensor information from SRS and predicting the user’s path. However, if the network quality of the WSNs in the location in which the user moves is unstable, the mobile device, in some instances, cannot receive the sensed data, and further, encounters the interoperability problem.

To address the interoperability problem under unstable WSN conditions, this paper proposes a SRS that uses network coverage information. By using the coverage information of the WSNs provided by OpenSignal [6], the network quality of the WSNs can be detected. If the network quality of the next position that is predicted by PP-SRS is determined to be poor, the proposed method can pre-fetch the sensed data information for that position from the SRS. Thus, the prevention of incidents or even crimes can be facilitated, even when the network is unstable. Additionally, this paper presents a novel method based on the probabilistic distribution of the users accessing SRS to detect the network quality of the next position, rather than OpenSignal. According to the experimental results, as compared to the existing PP-SRS, the proposed system shows an approximately 40% improved performance. This indicates that the proposed methods can be used to enhance various IoT-based services.

The remainder of this paper is organized as follows: Section 2 introduces the background. Section 3 present a SRS that uses network coverage information. Section 4 describes the implementation. Section 5 provides the experimental setup and results. In Section 6, related work is discussed. Finally, the concluding remarks and future work are summarized in Section 7.

## 2. Background

### 2.1. Sensor Registry Systems

A WSN is a physical infrastructure that is required for the Internet of Things (IoT) ecosystem. In a WSN, there exist a sink node and a group of sensors. The sink node is also a sensor node that collects and combines various types of sensed data (e.g., humidity, temperature, sound, pressure, motion, and vibration) from the sensors within the WSN. In this article, these sensed data are called instant data. The sink node regularly sends the instant data to the mobile devices of users who are in the vicinity of the WSN. Finally, applications running on the mobile devices provide the users with intelligent services through the mash up of heterogeneous instant data. 

To combine various instant data, the metadata of each instant data must be corrected. For example, the new terms are defined to be used by other applications. In addition, redundant metadata are removed and relevant metadata are semantically linked to the existing metadata in the Sensor Registry System. For this process, the metadata of each instance data are first submitted to the centralized Sensor Registry System. Then, a few metadata experts evaluate the submitted metadata, and make corrections, if necessary. In this way, the Sensor Registry System plays an important role in registering and managing the heterogeneous metadata of instant data sent by sensors.

A user’s mobile device continues to receive instant data collected by sensors whose purpose is to unceasingly measure a physical quantity for a particular event in real time. In parallel, the mobile device also receives metadata associated with the instant data from the Sensor Registry System. The metadata enables the mobile device to conduct semantically consistent interpretation of the heterogeneous instant data sent by a variety of sensors. In this article, we clearly assume that there are two physically different networks in the proposed scheme. One is a wireless sensor network, in which the mobile devices receive instant data sent by sensors. Through the other network, the mobile devices also receive metadata sent by the Sensor Registry System. Technically, the Sensor Registry System sends the metadata via Internet, and then the mobile device finally receives the metadata sent by a mobile station, which is the end-point of the Internet. In particular, we consider communication under bad conditions, where the mobile devices cannot receive any metadata because of the encountered communication problems. We also assume that the wireless sensor network is working in any case. Consequently, in the proposed system architecture, mobile devices receive instant data from sensors via the wireless sensor network and metadata associated with the instant data from Sensor Registry System via the Internet.

The Sensor Registry System has been developed by using the conceptual model based on ISO/IEC 11179. It registers and manages the heterogeneous metadata of instant data. In the real world, when a user is moving to another sensor network in the vicinity of the current sensor network, the Sensor Registry System sends semantically consistent metadata of the instant data to the user’s mobile device. Consequently, although the mobile device is transferred to different wireless sensor networks, it continues to provide service using instant data, overcoming the metadata interoperability problem, which commonly occurs in various sensors around different wireless sensor networks. For example, suppose that there are two sensor networks in Korea and the USA. A mobile device receives temperature information from thermometer sensors in Korea, where the sensors collect the information on the centigrade scale. Meanwhile, if the user travels to the USA, their mobile device will experience difficulty in processing temperature data from thermometer sensors in the USA. This is because all sensors measuring temperatures in the USA output the temperature using the Fahrenheit temperature scale. To address this metadata interoperability issue, a Sensor Registry System can retain semantically consistent metadata, indicating that Celsius is the same as Fahrenheit. If the user’s mobile device is operating in the USA, the Sensor Registry System will send semantically consistent metadata to the mobile device so that it continues to interpret temperature information without any limitation.

### 2.2. Path Prediction

The goal of the existing path prediction method is to predict a next desired segment among multiple candidate segments while a mobile device is moving along a path. For example, suppose there are paths like (a)→(b)→(c)→(d)→(e) and (a)→(b)→(c)→(d)→(f), where (a), (b), (c), (d), (e), and (f) are segments. If the current position of a mobile device is in the segment (d), the path prediction method can predict either (e) or (f) as the next suitable segment. The next position is determined by the greedy approach—e.g., (f) is determined if (f) was more frequently selected than (e) in the previous users’ routes. In the segment (d), the mobile device can prefetch metadata that are necessary for interpreting the instant data that will be sent by the sensors in the segment (f) when it arrives at the (f) segment.

In the proposed approach, extending the existing path prediction method, when a mobile device is moving to the segment (f) from the segment (d), it can forecast, using network coverage information, that the network will be not working in the segment (f) and mobile devices will not get any metadata in (f). Then, it can bring the metadata in advance, in order to interpret instance data in (f), using the prefetched metadata. In this work, as the network coverage information, the following information is employed:

- OpenSignal data includes a set of latitude, longitude, signal strength, telecommunication service provider(s). If the signal strength is greater than or equal to a certain threshold value (θ), the network in (f) is working in the local area (latitude, longitude). Otherwise, mobile devices cannot receive any metadata in the area.
- Previous log data in Sensor registry system—e.g., communication problems can be detected if the log data contains the frequent data transmission failure and retrials in the particular area of (latitude, longitude).

## 3. Network Coverage Information-Based Sensor Registry System for IoT Environment

This section introduces an extented SRS, named Network Coverage-based SRS (NC-SRS) that uses open network coverage data. PP-SRS suffers from a problem related to providing a seamless service. Non-service areas are areas where the mobile device using a SRS service experiences poor network conditions and does not receive the sensor information from the SRS. The sensor information is used for analyzing the data transmitted by the sensor device, for example, the identifier of the sensor and the unit of measure. When the sensor information does not received, the user cannot interpret the sensor data. The problem of continuous non-coverage is not be solved by using PP-SRS. Recent network support coverage information of the non-responding regions of a network can be obtained easily. When predicting the user’s path, it is possible to use the coveragence information of a network.

**Figure 1:**
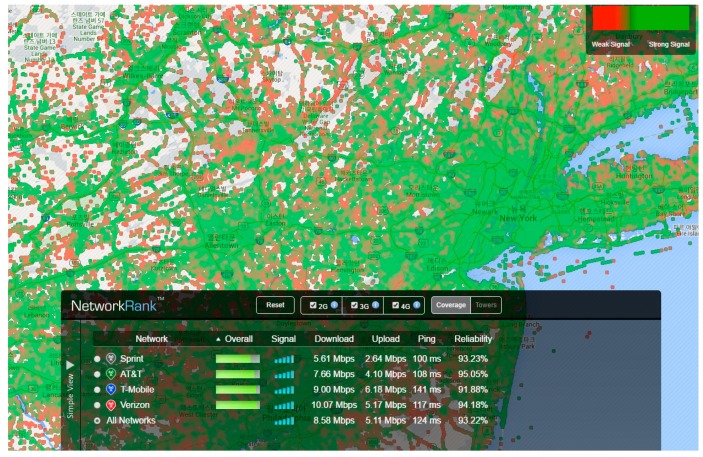
Example of network coverage data provided by OpenSignal.

Figure 1 illustrates the coverage information of a network provided by the OpenSignal application [6], which shows the signal strength corresponding to the regions on the map. This application shows the signal strength data of the carrier in a region having a predetermined range based on the GPS latitude and longitude information. The network coverage information includes the carrier information in the user’s area, communication bandwidth, signal strength, and upload/download speed. In this paper, to solve the problems related to an unstable network condition of PP-SRS, a method that uses the network coverage data has been proposed. PP-SRS is used for predicting the movement path of the user, when the mobile device has received the sensor information of the next path in advance. However, in a continuous poor communication area, the system does not respond. The mobile device utilizes the network coverage data and can recognize the network status of the user’s next path. The proposed method utilizes the coverage information and receives the sensor information of the poor communication area, in advance. The information of the mobile device’s carrier and communication type is the basic information required for creating the SRS poor communication group.

**Figure 2:**
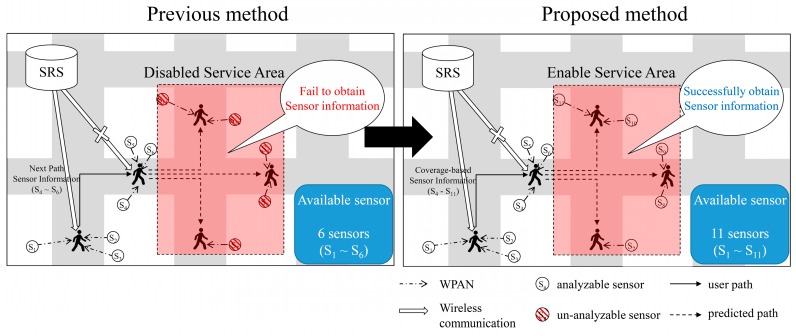
Network coverage information-based method overview.

**Figure 3:**
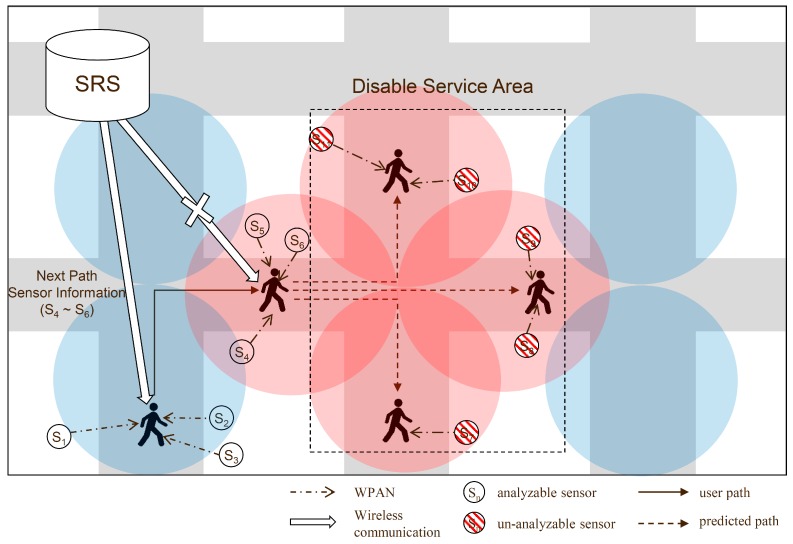
Example of sensor information received using path predict.

Figure 2 shows an overview of the proposed method. Figure 3 shows an example of the receipt of the sensor information by the path prediction-based system. The system receives the sensor information, in advance, before the user’s arrival at a position. If the prediction is successful irrespective of the communication state of the next path, the user’s mobile device receives the metadata using the previously received metadata, the sensor data can be interpreted. In the first path (1), when moving toward the next path (2), the device receives the sensor information of the next path, i.e., the information of sensors S_4_–S_6_. 

**Figure 4:**
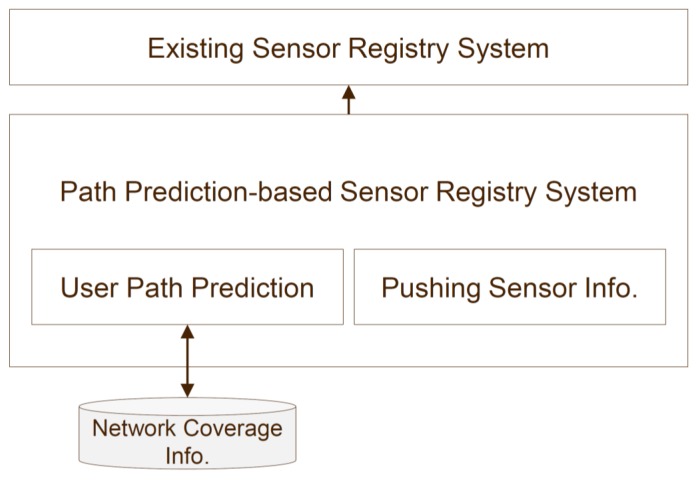
Concept diagram for extending PP-SRS.

When the communication state of the next path is poor, the mobile device uses the sensor information received in advance and analyzes the sensor data. However, if the coverage is poor in a continuous path, denial-of-service areas are formed, that is, areas where the service is disabled, which cannot be considered in the analysis of the sensor data. Sensors S_7_ to S_11_ in the disabled service area cannot interpret the received sensor data. To solve this problem, the proposed method uses the coverage information of the network to transfer the sensor information of the denial-of-service area continuously. Figure 4 presents concept diagram for extending PP-SRS.

**Figure 5:**
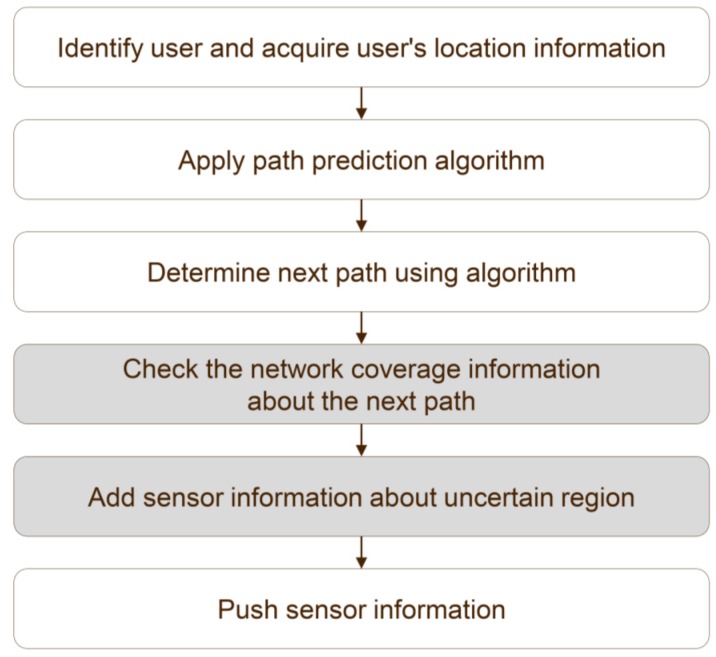
Overall procedure for providing sensor information.

Network coverage-based SRS provides users with sensor information on the basis of the coverage information of the network. In the proposed method, the user’s device must identify, in advance, the sensors that are in use, to obtain the necessary sensor information in advance. The existing path prediction-based method utilizes the user’s path that it has received, to predict the information for the next path. The proposed method evaluates the network coverage information in the next path, depending on the circumstances, and sends relatively rich sensor information. Figure 5 shows an application of the conventional path prediction-based system to obtain the information of the overall sensor data. The path prediction algorithm is applied to determine the predicted path. An additional process is proposed wherein the network coverage information for the predicted path is checked. The system utilizes the results of the predicted path and then checks the coverage information of the local network of the mobile device. The network coverage information used in the proposed method includes: (1) carrier information, i.e., SK Telecom, Olleh, or LGT; (2) signal strength, i.e., Received Signal Strength Indicator (RSSI) or Reference Signal received Poser (RSRP); and (3) information related to the network type, i.e., 2G, 3G, or 4G. By using the basic information and the coverage information of the network of the user’s mobile device, it is possible to prepare in advance the communication status of the movement path. For example, in the case of an unstable 3G signal, the information related to the network type of the mobile device is the signal strength of the 3G network in the area of the predicted movement route. If it is −80 dBm or less, the sensor information is sent in advance before the user’s arrival in that area. If the mobile device is unstable or has a weak signal strength, the sensor information is pre-loaded to provide seamless sensor data analysis in difficult environments.

**Figure 6:**
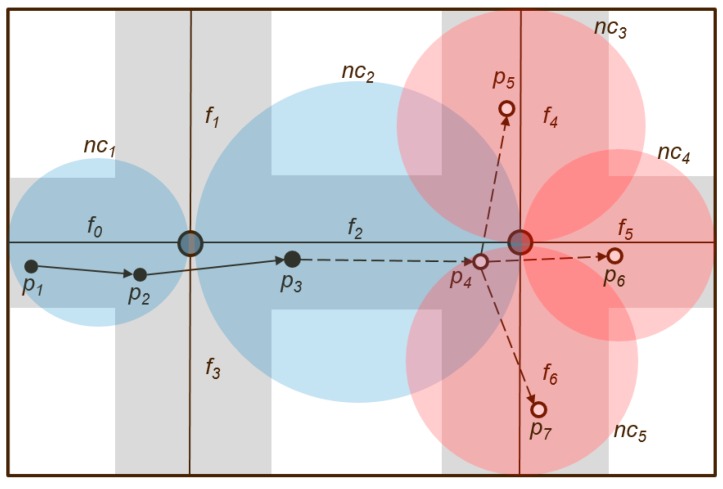
Graphical representation of applying path prediction.

When a path is fragmented by road intersections, the proposed method utilizes the fragments of the path, i.e., path segments. Figure 6 shows an example of the use of the network coverage when a path is predicted using the structure of the path segments. Here, *p_i_* denotes the user in a continuous position, the grayscale region represents the road along which the user is walking, *f_i_* denotes a path segment, *nc_i_* denotes the network coverage corresponding to the path fragment, the blue color indicates a good network state, and the red color denotes an unstable network state. Users *p*_1_, *p*_2_, and *p*_3_ sequentially move to *f*_0_ and *f*_2_. Their subsequent future paths to other locations are predicted: {*p*_4_ → *p*_5_, *p*_4_ → *p*_6_, *p*_4_ → *p*_7_}. The existing path prediction-based SRS, after the user navigates to *p*_4_, either predicts a move to any location in {*p*_5_, *p*_6_, *p*_7_} or sends the sensor information for that path. However, in the case of *p*_4_, because the network state is unstable in that area, the mobile device does not receive the sensor information from the server, and thus, cannot interpret the received sensor data. The proposed method checks the network coverage state of the path fragment at the server in advance. If the user is expected to move along a path fragment where the network is unstable, the information of the sensor corresponding to {*nc*_3_, *nc*_4_, *nc*_5_} is sent when the user is at position *p*_3_. The mobile device, regardless of the status of the network in the *p*_4_ area, can interpret the peripheral sensor data. When the mobile device is moved to a normal network region, it can utilize PP-SRS.

**Figure 7:**
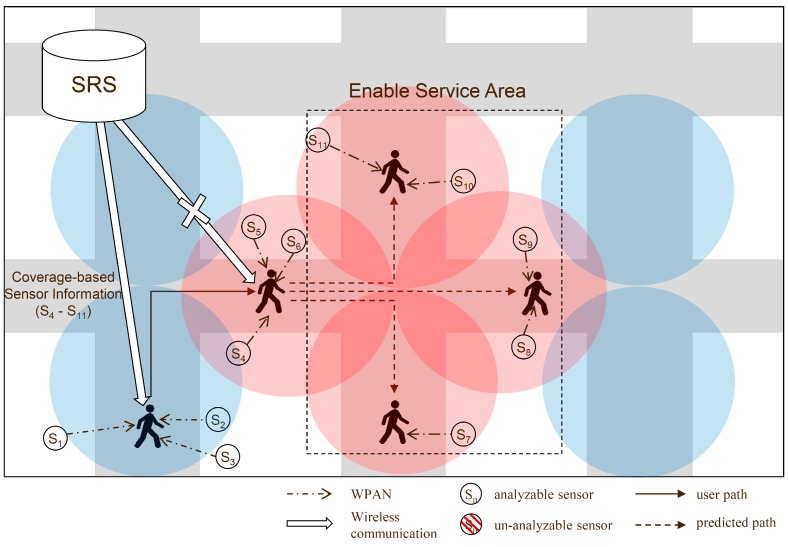
Example of sensor information received using coverage information.

Figure 7 shows an example of the sensor information received on the basis of the coverage information provided. A service reception denial region is formed to solve the problem and to extend PP-SRS. The coverage state depends on the carrier and the type of network, 3G or LTE. By utilizing the functionality provided by OpenSignal, the signal strength of a mobile device in a desired region can be obtained. The system saves the signal intensity corresponding to the type of network of each carrier in the path segment, and by using this information, it determines the area where local services cannot be received. All the sensor information of the next path, which is in an area where the signal strength is weak and is regarded as a disabled service area, is sent, including that of sensors S_4_–S_11_. The user’s mobile device may interpret the sensor data received from the peripheral sensors in the reception-disabled region of the service. Thus, it is possible to reduce the loss of raw data.

Algorithm 1 shows a major portion of the measurement algorithm of a denial-of-service area mentioned in the pseudo-code. Using the algorithm, a set of path segments in a denial-of-service region can be determined. In Algorithm 2, a depth first search (DFS) is used to group the connected path segments. SRS sends the sensor information of all the path fragments in the denial-of-service area on the following path of the mobile device.

**Algorithm 1.** disabled_coverage_measuring (network type, vendor, standard_strength)
**Input:** network type of user mobile phone, vendor of user mobile phone, standard_strength of disable signal standard
**Output:** set DC[] of disabled segments1:Segment [] ← get all segments ()2:**for each**  Segment:s  **do**3:  cp ← get_center_point (s)4:  sd ← get_distance (s)/25:  ss ← get_signal_strength (cp, sd, networktype, vendor)6:  sec ← get_SRS_enable_check (s)7:    **if**   ss < standard_strength  or  sec = True  **then**8:       DC[] ← s9:    **endif**10:    **if**   sec = False   **then**11:        Remove s from DC[]12:    **endif**13:**endfor**14:**return** DC[]

**Algorithm 2.** disabled_coverage_grouping (DC[], G)
**Input:** set DC[] = {s1, s2, …, sm} of disabled segments, a segment graph G
**Output:** set S[] of disabled group1:Let S[] be a stack2:Segment[] = DC[] //get all disable segment3:n ← 0  //group number4:**while** Segment[] is not empty5:    S[n].push(Segment[0])6:    Remove Segment[0] from Segment[]7:    **while** S[n] is not empty8:        v = S[n].pop()9:       **for**
**all** edges from v to w in G.adjacentEdges(v) **do**10:            **for each** segment:s **do**11:               **if**     Segment[s] = w  **then**12:                   S[n].push(w)13:                   Remove Segment[i] from Segment[]14:                **endif**15:            **endfor**16:       **endfor**17:    **endwhile**18:    n ← n + 119:**endwhile**20:**return** S[]

## 4. Implementation

To utilize the proposed PP-SRS, an application running on the server and mobile device was implemented. A track and path prediction application that displays the user’s location was developed. Table 1 shows the development environment.

**Figure 8:**
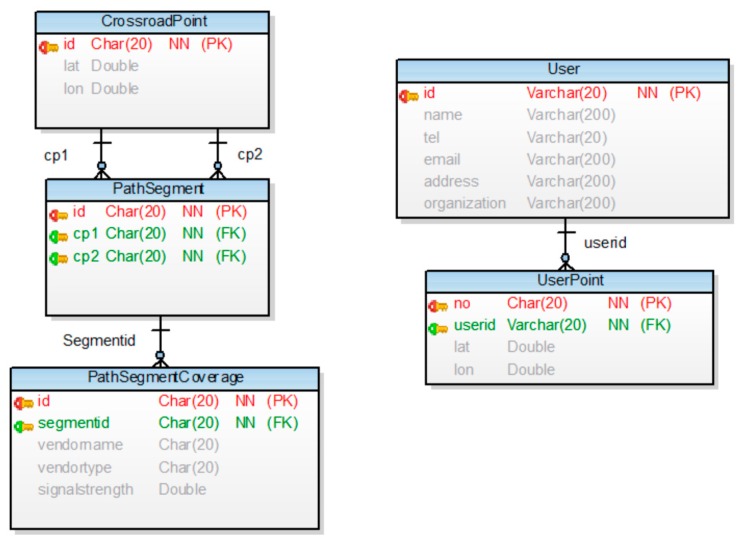
Data model of network coverage-based path prediction.

**Figure 9:**
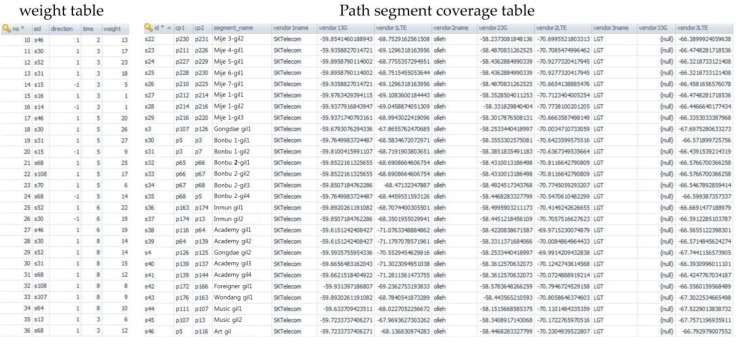
Snapshot of implementation using network coverage information.

**Figure 10:**
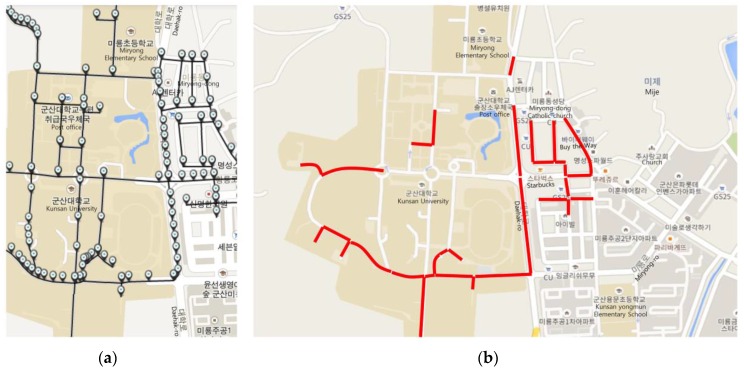
Snapshot of implementation: (**a**) Intersection points and path segments; (**b**) example of service disabled path segment.

Figure 8 shows a data model of the network coverage algorithm. The *User* table is used to identify the user. The *UserPoint* table saves the user’s location and uses it for tracking purposes. The *Intersection* and *PathSegment* tables are used to represent the road. The *PathSegmentCoverage* table is used to store the coverage information of the networks; this table, together with the *PathSegment* table, stores the carrier type of the mobile device communication and the signal strength. Next, Figure 9 shows network coverage data of the path fragments and weight data of CBP. The path is located inside and nearby Kunsan National University. The coverage information consists of OpenSignal information. Carrier type LTE is composed of SK Telecom, Olleh, and LGT. Carrier type 3G is composed of Olleh. Figure 10 shows a screenshot that captures the implementation results of the proposed method. Figure 10a shows the intersections of the path fragments collected from the mobile device. The server that receives the location information of the user’s mobile device identifies a nearby path fragment. Figure 10b shows the coverage information on the server, indicating the denial-of-service areas. The server identifies the denial-of-service service regions and receives the mobile device information, i.e., the carrier and communication type. Continuous denial-of-service areas are grouped using the DFS search algorithm.

## 5. Experimental Results

This section shows that the proposed NC-SRS method based on Algorithms 1 and 2 described in Section 4 is more accurate than the existing PP-SRS method. It is worth noting that PP-SRS method is likely to significantly reduce the accuracy of the task of predicting a path if the network status is partly unstable along the path. On the contrary, the proposed NC-SRS is a robust method even in many of the cases in which the quality of the network is not sufficiently good to enable a mobile device to continuously obtain metadata from the Sensor Registry System. Obviously, obtaining proper metadata at a time is very important to the mobile device. While the mobile device is moving along a predicted path, it is always receiving instant data from sensors via a wireless sensor network. However, unless it has the very metadata corresponding to the instance data, it cannot grasp the meaning of the instant data using the metadata. The proposed method can detect the parts of a path in which the communication problem might occur, in advance. The proposed method works based on the use of open signal data and the mining of the provenance information of the sensing registry system (see Algorithm 1). If it is detected that there might be disconnected areas in a predicted path, then the method attempts to obtain the metadata from the Sensor Registry System, in advance. In this way, the mobile device can seamlessly interpret instance data sent by sensors, using the relevant metadata sent by Sensor Registry System.

**Figure 11:**
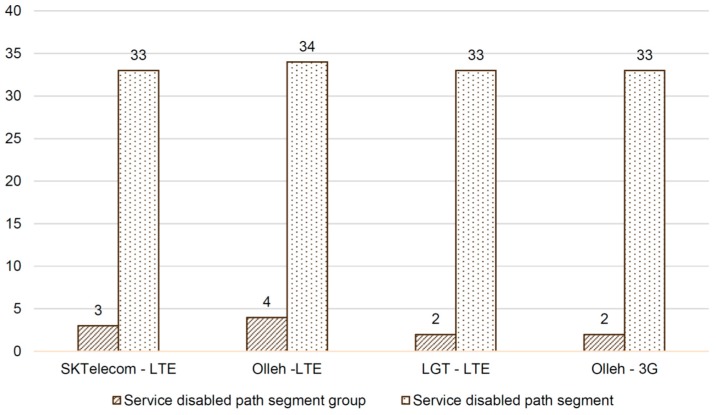
Service disabled path segment and group size evaluation result.

To evaluate both PP-SRS and NC-SRS methods, 21 undergraduate students in the Kunsan National University participated in the experiment. The moving route data of the students were gathered for 15 weeks. The raw data, such as date, time, and GPS co-ordinates, were collected from the smartphones of the 15 students. It was concluded that each student tended to have 4~5 paths per day on an average. To obtain the real paths, the raw data were projected on the campus map. In the data set, the number of total paths is 489. In addition, a path consists of several segments. At a fork in the road, there are several segments to move. The number of total path segments is 117. Figure 9 and Figure 10 illustrate these details. Figure 11 shows basic statistics, indicating the counts of the disconnected path segments of the major three public telecommunications business operators in Korea—SK Telecom, LG Telecom, and KT (Model name: Olleh). For the experiment, the network coverage information of the three companies is collected from the OpenSignal website. The network types are 3G and 4G (LTE). To determine whether a path segment is disconnected or not, the specific signal strength threshold value for each carrier is defined by domain experts. For example, the threshold values are {SK Telecom, LTE, −85.2}, {Olleh, LTE, −83.6}, {LG Telecom, LTE, −83.2}, and {Olleh, 3G, −74.6}. Overall, the number of the unavailable path segments is 33 on an average. The rate of the unavailable path segments is $\frac{33}{117}\times 100=28\%$. In addition, the areas of continuous disconnected path segments are defined as the service disabled path segment groups. The number of the groups is three on average. Moreover, each group is composed of three or four continuous path segments. In the worst case, it has at most six path segments. Figure 11 is a good motivated example of the research problem presented in this article. In this real environment, it commonly appears that the existing PP-SRS method does not working effectively owing to the existence of several disconnected segments in the predicted paths.

**Figure 12:**
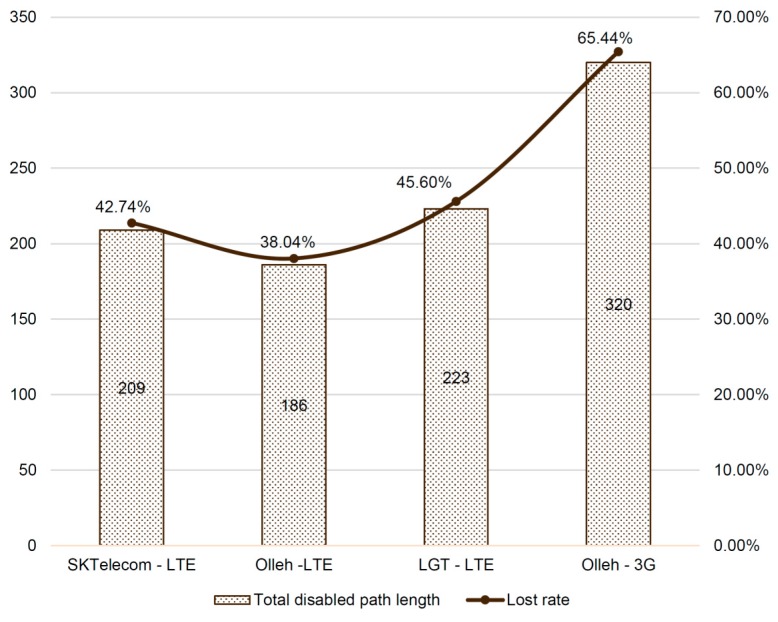
Total disabled path length and loss rate evaluation result.

Figure 12 depicts the number of paths, each of which has at least one segment with network service failure. Interestingly, the different vendors tend to have considerably different numbers of paths. For example, SK Telecom has about 43% of the total paths, each of which has at least one disconnected path segments. Specifically, 209 paths contain at least one disconnected segment among the total 489 paths. Meanwhile, KT and LG Telecom have approximately 38% and 46% of the total paths, respectively. In case of 3G, the result is poorest—e.g., about 65%. This means that network quality is different for different companies and the network quality of LTE (4G) is much better than that of 3G.

**Figure 13:**
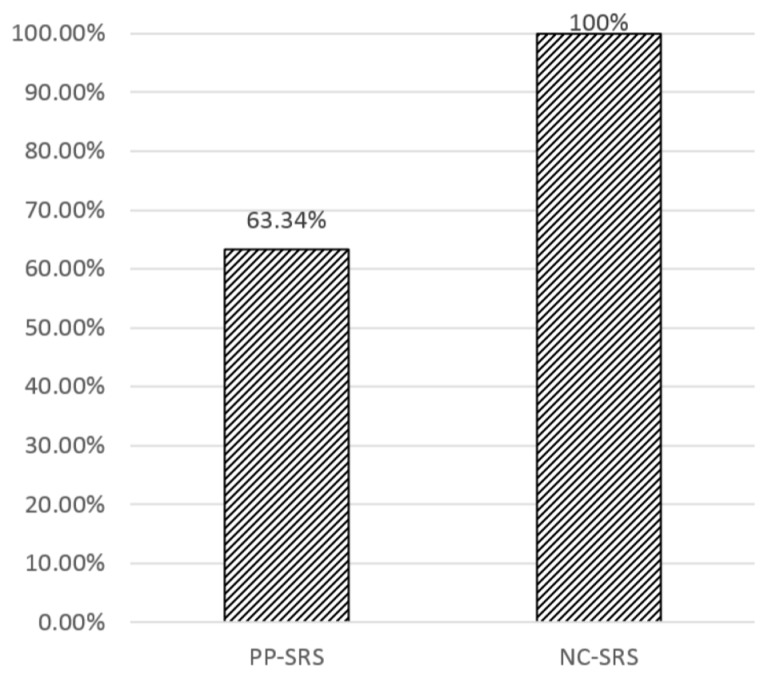
Service provision rate of PP-SRS and NC-SRS.

**Figure 14:**
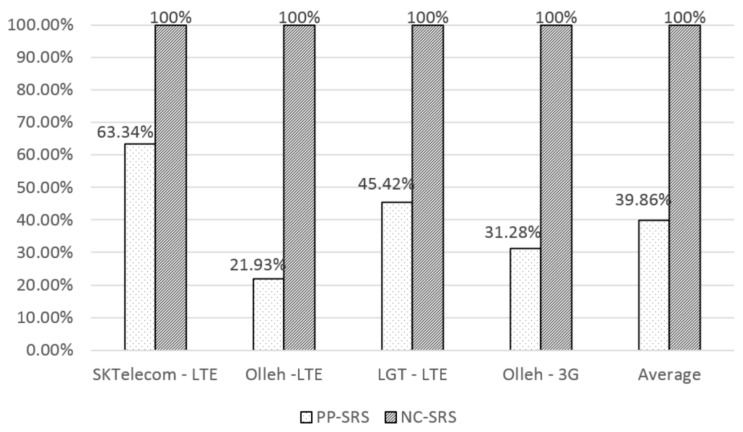
Comparison of the service provision rates of each carrier using PP-SRS and NC-SRS.

Figure 13 shows a service provision rate of the existing PP-SRS method and the proposed NC-SRS method. From the total of 489 paths, ten-fold data set were made first, each of which contained nine training sets and one test set. The service provision rate is computed by $\frac{\#\text{ of provided service}}{\#\text{ of service requests}}$. To measure the rate, we assume the prediction accuracy is 100% for both of PP-SRS and NC-SRS because NC-SRS method only considers whether services are successfully provided or not. SK Telecom is selected as a network service for the experiment in Figure 13 because SK Telecom provides the best network service as compared to LG Telecom and KT as shown in Figure 14. NC-SRS discovers the unavailable network service areas along the predicted paths; the proposed NC-SRS method will obtain the metadata from the Sensor Registry System, in advance. Thus, even though a mobile device is moving in the area under network service failure, it is able to seamlessly process instant data sent by sensors, using the relevant metadata sent by the Sensor Registry System. In contrast, since PP-SRS does not have any intelligent process of detecting the disconnected parts of paths, it can no longer interpret instant data sent by sensors. In Figure 13, PP-SRS shows about 63.34%, while the proposed method indicates 100% for service provision rate. This experimental result means that the proposed NC-SRS method does not have any limitation of processing instant data in the environment where there are communication problems.

Figure 14 illustrates the service provision rate of both PP-SRS and NC-SRS for the different communication companies in Korea. The proposed NC-SRS method shows 100% because a mobile device using NC-SRS has received metadata in advance before it enters unavailable network service areas along the predicted path. In this way, the mobile device cannot lose necessary metadata in the circumstance of unavailable network service. However, the existing PP-SRS method can show very poor rates for different communication vendors. For instance, the service provision rates of PP-SRS in SK Telecom, KT(LTE), LG Telecom, and KT(3G) are 63%, 22%, 45%, and 31%, respectively. Expectedly, the patterns of PP-SRS are very similar to the rates of paths with network failure by the vendors, as shown in Figure 13. The average rate of PP-SRS is up to 40%.

In summary, there are two major findings of the experiments. First, in actual applications, to which the existing path prediction methods are applied, a number of paths turn out to contain considerable segments where a mobile device cannot obtain metadata from a Sensor Registry System, which is necessary to interpret the meaning of instant data sent by sensors. These experimental results strongly support the reason why the proposed NC-SRS method is required in the traditional path prediction problem.

Second, the service provision rate of the existing PP-SRS method is up to 40% across the major communication vendors in Korea. However, the proposed NC-SRS method is able to seamlessly process instance data, using metadata in mobile devices. In the area where the network status is unstable and even metadata is not sent to the mobile device, NC-SRS method receives the proper metadata from the Sensor Registry System in advance, using (1) OpenSignal data and (2) the previous log data of the Sensor Registry System. Consequently, without affecting the limitation of physical network status, the NC-SRS method can enable mobile devices to cope with the instant data sent by sensors, to eventually provide seamless service to the end-users of mobile devices.

## 6. Related Work

In this section, we discuss SRS and network coverage information that were employed for understanding SRS and mobile crowdsensing.

### 6.1. Sensor Registry System

The network conditions of the existing SRS are unstable and thus they do not reliably provide sensor data. To solve this problem, enhanced SRS was developed based on path prediction [4]. This path prediction-based SRS can predict the moving path of the user to pre-load the sensor data of their future position into a mobile device. It can also reliably process and interpret sensor data under unstable network conditions. This study applies the weight of the moving path for each time period to improve the path prediction performance. 

However, the path prediction of SRS under unstable network conditions has certain limitations. SRS predicts the movement path of the user’s mobile device and transmits the relevant sensor information. However, when the predicted travel path is not within the coverage area of the network, a mobile terminal may not be able to receive the sensor information, as in areas not included within the network coverage. The connection between the mobile terminal and sensor registry management system becomes disabled. Thus, the mobile device does not receive the sensor information required to process the received sensor data and the quality of the associated application service is therefore degraded. 

In the method proposed in [7], the user’s movement route data, distance, and time are collected, and then, the degree of similarity of the path with other frequently used paths is measured using the direction element. The method then selects the highest path similarity. The study focused on the fact that destination prediction is impossible if no past movement route information for the user exists, and therefore, the method is not suitable for short-distance movement path prediction. 

Using the existing mobile path prediction methods, which focused primarily on the prediction of a personalized travel route, it is impossible to achieve a prediction in the absence of past movement route data. Further, since the object of the prediction is the final destination, the methods are not suitable for short-distance travel path prediction. In particular, a fast response speed to a request in a real-time processing environment is required. Fast movement prediction algorithms for short-range users that can overcome these limitations, such as the Collective Behavior Pattern (CBP) prediction algorithm, have been proposed [8]. CBP uses the greedy strategy concept to provide a high-speed processing performance. For this reason, fragmented paths are managed on the basis of the intersection of the road. A fragmented path is defined as a path fragment set. The path fragment has a direction and a weight. By selecting the path fragment with the highest weight from all the path fragments, the movement route can be predicted.

### 6.2. Network Coverage Information

Network coverage information is mobile cellular network information based on mobile crowdsensing. The mobile crowdsensing approach turns user mobile phones into sensing devices with measurement software running in the background. This approach has the advantage of enabling cost-effective, fine-grained and continual spatiotemporal wireless monitoring [9]. 

OpenSignal [6] and Mobiperf [10] represent crowdsourced mobile network measurement systems with website and mobile apps. OpenSignal provides the quality of mobile networks such as the available name of the network, the average RSSI in Arbitrary Backbone Unit (ASU) and dBm, the average download speed in kbps and the average ping time in milliseconds. The quality information can predict the user network condition with GPS information, network name, and network type. 

Urban WiFi characterization [11] presents a mobile crowdsensing approach that leverages commodity smartphones and the natural mobility of people and monitoring through a measurement study in the city of Edinburgh. This method indicates the WiFi source in practice at locations with high AP densities. MCSSENSE [12] describes a full-featured geo-social crowdsensing platform for smart cities. This platform automates the organization of spontaneous collaborations of large groups of people participating in collective actions, such as in the notable case of urban crowdsensing. MCS [13] use the power of citizens for large-scale sensing. MCS goes beyond participatory sensing by having implicit and explicit participation, and the collection of crowdsourced data from both mobile sensing and mobile social network services. CAROMM [14] presents a mobile crowdsensing system and focused on developing an efficient and scalable data collection model that aims to reduce energy and bandwidth consumption related to continuous sensing and uploading in such applications. TRAC [15] have investigated designing incentive mechanisms for stimulating smartphone users to join mobile crowdsourcing applications with smartphones. TRAC have considered the crucial dimension of location information in the design of incentive mechanism. 

Kiljander et al. [16] addressed the interoperability problem of IoT devices that tend to create and combine heterogeneous metadata. They proposed a semantic-level interoperability architecture that utilizes IoT and pervasive computing devices. To resolve heterogeneous metadata, IoT devices are expressed as ontology through the semantic web knowledge representation function of the semantic information broker in the approach. Furthermore, FIESTA-IoT [17] extended the basic metadata model of [16] and presented experimental results using the experimentation-as-a-service (EaaS) paradigm. Similar to Kiljander et al.’s approach, IoT devices are represented as a standards-based ontology concept and users can access desired metadata using EaaS application program interface. However, both [16] and [17] do not describe the detailed method of real applications. Unlike NC-SRS in this article, the existing methods do not make seamless service for users that encounter the network connection problem. Table 2 summarizes the comparison of NC-SRS with the existing methods [16,17]. Our work has focused on disabled coverage measuring and grouping mechanism for seamlessly providing services.

## 7. Conclusions

This paper has proposed an extended SRS capable of providing sensor information stably in areas that are not supported by a network or where the network coverage is weak. Sensor information needs to be received seamlessly, because it is used for interpreting the data received from the various sensors. In other words, the provision of reliable sensor information to the user is essential for providing a stable service. Therefore, a method that predicts the moving path of the user and sends the sensor information to the user’s mobile in advance, using open network coverage information was presented. The open information of the mobile terminal and the user’s network coverage, that is, the local telecommunications company’s information, signal strength, and network type, are checked, and the sensor information is provided in advance. In the proposed method, as compared to the existing path prediction method, the mobile device receives additional sensor information according to the network state. An additional overhead is incurred by including the sensor information for continuous regions. The user’s quality of service is relatively less influenced by the state of the network, and the user’s mobile device is capable of processing continuous sensor data. The operation of users’ mobile devices is stable, because they can process the sensor data, thus, they enabling the provision of a stable quality of service. In future, the impact of the overhead generated by adding sensor information on performance will be investigated.

## Figures and Tables

**Figure A1:**
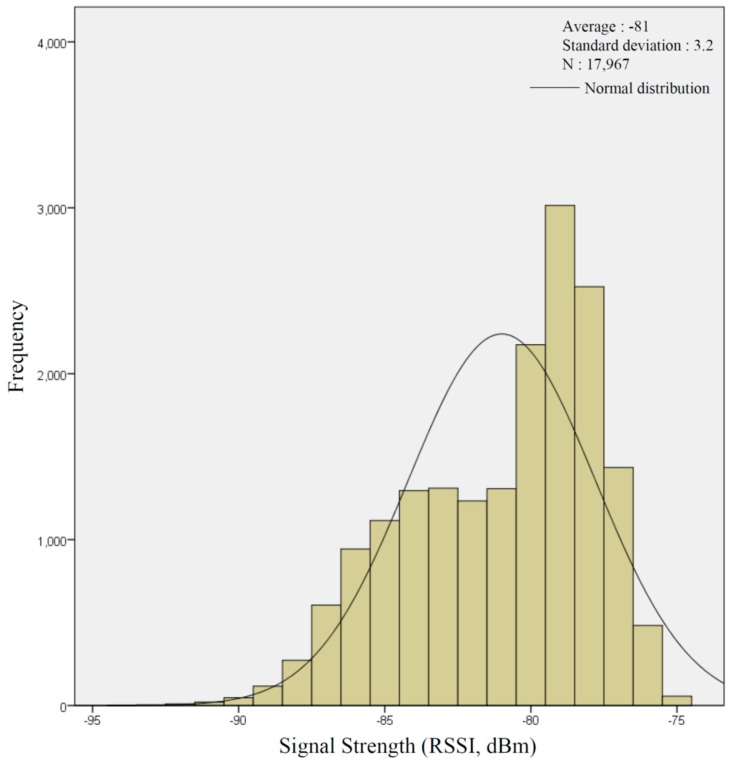
Histogram of the frequency of total signal strength.

**Figure A2:**
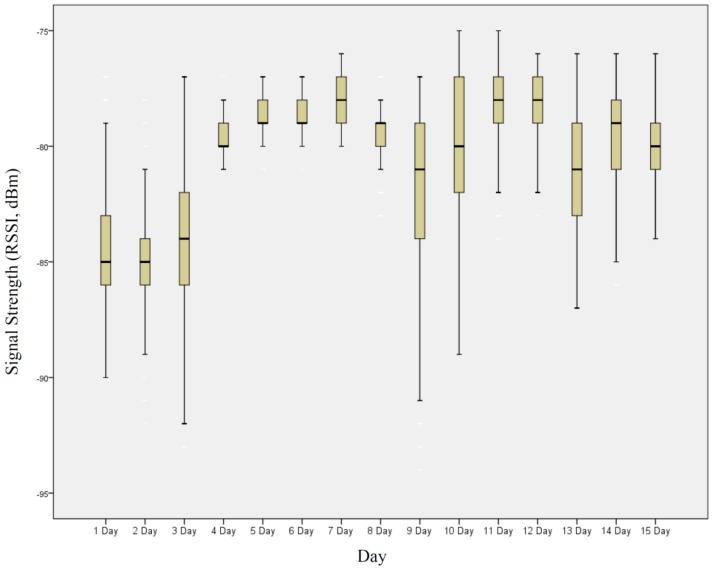
Box plot of signal strength for each day.

**Figure A3:**
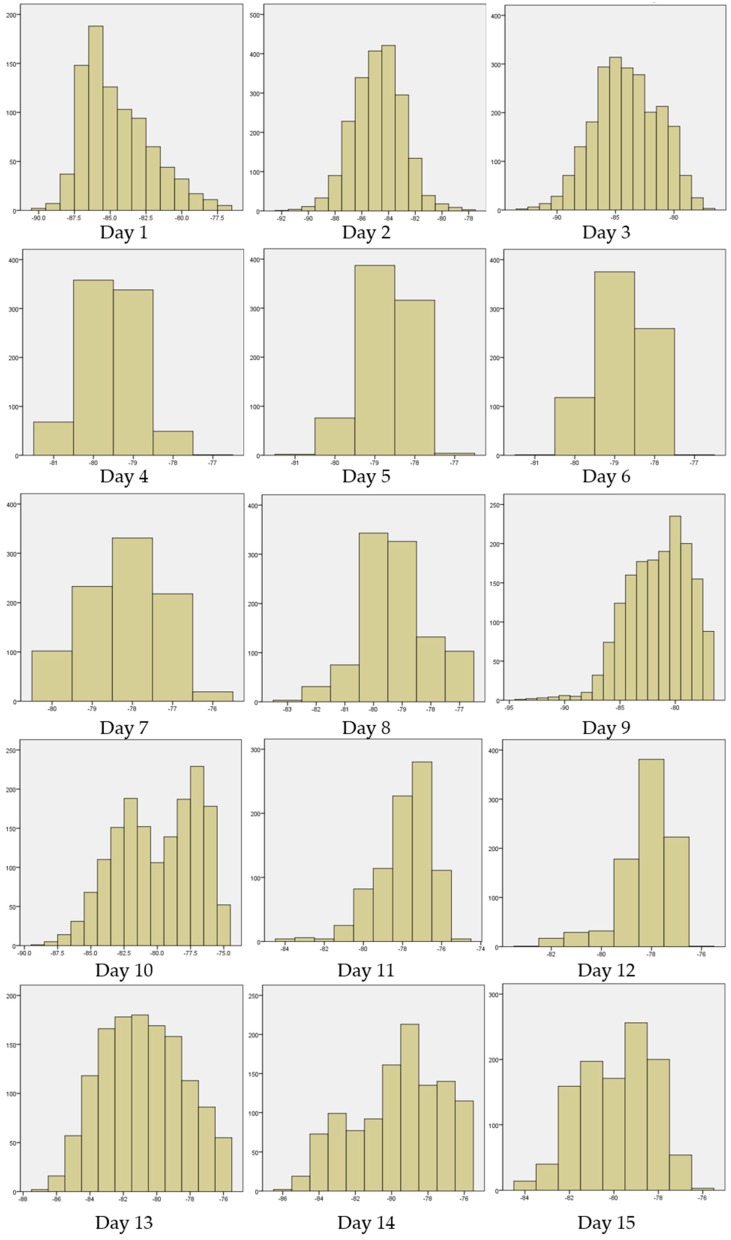
Histogram of signal strength on each day.

**Table 1:** Development environment.

Feature	Details
Operating System	Windows 7 Professional K (×64)
Processor	Intel(R) Core(TM) i5-3570 3.80 GHz
RAM	12 GB
Development Language	Java
Mobile OS	Android OS
Android Emulator Version	4.1.2
Web Server	Apache Tomcat 8.0.8
Database	MySQL 5.5

**Table 2:** Comparison of NC-SRS with the existing methods [16,17].

	Similarities	Differences
NC-SRS vs. Kiljander et al. [16]	➀ Both researches assume the same physical environment as heterogeneous IoT environment. ➁ Both methods propose the metadata model for managing heterogeneous metadata for IoT devices, using MetaData Registry (MDR) concept. ➂ Both methods provide similar metadata model for sensing device, location, actuators, and unit of measure.	➀ NC-SRS implements the metadata model through Entity Relationship-based model but Kiljander et al. make use of ontology-based model. ➁ NC-SRS method is based on ISO/IEC 11179-based registration and management of heterogeneous metadata but Kiljander et al. never consider it. ➂ The applications of both methods are different. NC-SRS is working in emergent situations but Kiljander et al. consider smart space applications. ➃ The most distinct point of the two methods is: Kiljander et al. focus merely on defining a ontology-based metadata model for managing heterogeneous metadata. However, NC-SRS supports seamless services for users with network connection problem, using network coverage information. Our method is working on top of Kiljander et al.’s method.
Kiljander et al. [16] vs. FIESTA-IoT [17]	➀ FIESTA-IoT extends the basic functions of Kiljander et al.’s metadata model.	➀ FIESTA-IoT manages heterogeneous metadata in the same way as Kiljander et al.’s method. Moreover, FIESTA-IoT adds more detailed ontologies and service definitions. ➁ Unlike Kiljander et al.’s method, data exchange model is additionally defined using JSON. ➂ FIESTA-IoT newly presents experimental results including case study of real applications.

**Table A1:** One-way variance analysis.

Signal Strength					
Source of Variance	Sum of Squares	Df	Mean Square	F	*p* Value
Between Group	101,819.299	14	7272.807	1588.736	<0.001 *
Within Group	82,179.436	17,952	4.578		
Total	183,998.735	17,966			

* *p* < 0.001: Highly Significant.

## Appendix A. Statistical Hypothesis Test

In this study, it was assumed that the signal strength at a location is not constant. According to the OpenSignal site, the signal strength does not change over time. However, it has been shown empirically that the signal strength at a location constantly changes. A statistical hypothesis test was conducted to verify this. The signal strength of a mobile device using a Samsung Note II and LGT carrier was measured. The changes in signal intensity were measured for 15 days at one location. The hypotheses of this paper are:

Null hypothesis (H0): The standard deviation of 15 days is zero.

Alternative Hypothesis (H1): The standard deviation of 15 days is not zero.

The adopted significance level was 95%. Table A1 shows the results of the one-way analysis of variance. The p-value, which is less than the significance level, is sufficiently weak to support the null hypothesis, or is sufficiently strong to reject the null hypothesis, that is, to adopt the alternative hypothesis.

Figure A1 shows a histogram of the frequency of total signal strength. The signal strength changed 17,967 times in 15 days. The average value is −81 dBm. The standard deviation value is 3.2 and the distributed value is 10.241. The maximum value is −94 dBm and the minimum value −75 dBm. Although the mobile device was not moved during the measurements, the results show that the signal strength for each date and time varies. The variance value of 10.241 shows that a change occurs in the signal strength. That is, the numerical value can change from Good to Excellent or from Fair to Poor.

Figure A2 and Figure A3 show the signal strength for each day. Although the signal strength would be expected to be constant at a location, it can be seen that the change between the days is significant. In addition, the signal strength is not constant during the day, and large changes occur. By using an application such as OpenSignal, when the signal strength is average, it is possible to determine the signal strength of the region. However, there is a possibility that the signal strength for each day is changed.
